# Gestational and Neonatal Outcomes in Cities in the Largest Coal Mining Region in Brazil

**DOI:** 10.3390/ijerph191912107

**Published:** 2022-09-24

**Authors:** Renata Dupont Soares, Marina dos Santos, Fernando Rafael de Moura, Ana Luiza Muccillo-Baisch, Paulo Roberto Martins Baisch, Maria Cristina Flores Soares, Flavio Manoel Rodrigues da Silva Júnior

**Affiliations:** Institute of Biological Sciences, Universidade Federal do Rio Grande—FURG, Av. Itália, Km 8, Campus Carreiros, Rio Grande 96203-900, Brazil

**Keywords:** maternal exposure, newborn, prenatal care, environmental pollution, Candiota

## Abstract

Brazil has one of the largest mineral coal reserves in the world. More than 40% of this ore is in the Candiota Mine, in the extreme south of Brazil, which was previously identified as a hotspot of environmental pollution. In addition, an important part of Brazil’s population suffers from socioeconomic vulnerability. Since there is no information on unfavorable gestational and neonatal outcomes associated with these problems, we conducted a cross-sectional study with 1950 mother–child binomials, aiming to evaluate the association between these outcomes and air pollution as well as socioeconomic, demographic and health variables in seven cities in the region. Of the total births, 11.6% were preterm and 9.5% of neonates had low birth weight (<2500 g). These conditions were also associated with skin color, previous abortions, birth type and prenatal care, as well as exposure to higher levels of coarse particulate matter (PM_10_) during the first trimester of pregnancy. Regarding air pollutants, although the daily limits for PM_10_ were exceeded on less than 5% of days, the annual average overtook the values proposed by WHO. Thus, we concluded that prematurity and low birth weight in this region are related to air pollution, and to socioeconomic variables and health care.

## 1. Introduction

Coal is the most harmful energy source for the environment [1] because the products of coal combustion contain inorganic chemicals such as mercury, sulfur, fluorine, lead, and arsenic, as well as organic molecules including aliphatic hydrocarbons, polycyclic aromatic hydrocarbons (PAHs), alcohols, carboxylic acids, aldehydes, ketones, and aromatic nitro compounds [2,3], which cause significant health effects [3,4].

Developing countries have encouraged the policy of using coal for power generation based on increased demand for energy and the economic benefits generated [5]. Brazil is among the countries with the largest mineral coal reserves, and the largest national reserve is located in the southern region of the country. The Candiota region is regarded the most important coal mine in the country, accounting for about 40% of the national coal reserve [6,7,8], in this region there is one coal-fired power plant and two coal surface-mining operators [9,10]. Candiota coal is of low quality; consequently, the emission levels of atmospheric pollutants are higher than those from similar plants elsewhere [11,12].

Coal exposure has several negative impacts on human health, especially in vulnerable groups such as pregnant women and children. Maternal exposure to ambient air pollution is a well-recognized risk factor for adverse neonatal and obstetric outcomes, due to the significant transplacental transfer of contaminants [4,13,14]. Additionally, exposure to coal pollutants in that period can result in irreversible damage to maternal and child health [15]. Fetal growth restriction, gestational maturity, low birth weight, preterm delivery, birth defects and neonatal morbidity and mortality have been reported as related to coal mining [13,16,17,18,19].

The public health impact of coal-based energy production on maternal and child health is a concern worldwide, especially considering the elevated cost to public health of environmentally mediated diseases [20]. Coal pollutants impact the entire population—both local and nearby communities—both directly and indirectly [13,17,18,19,21].

Research in the study region has shown an environment with moderate levels of contaminants [11,22], but these levels already pose a potential risk to human health [23,24,25,26]. Epidemiological studies have shown damage to health from exposure to coal [27,28,29,30], but the information on maternal and neonatal outcomes is still scarce in Brazil. Thus, the aim of this epidemiological study was to analyze maternal and neonatal outcomes in the largest coal mining region of Brazil.

## 2. Materials and Methods

### 2.1. Study Area

**Figure 1:**
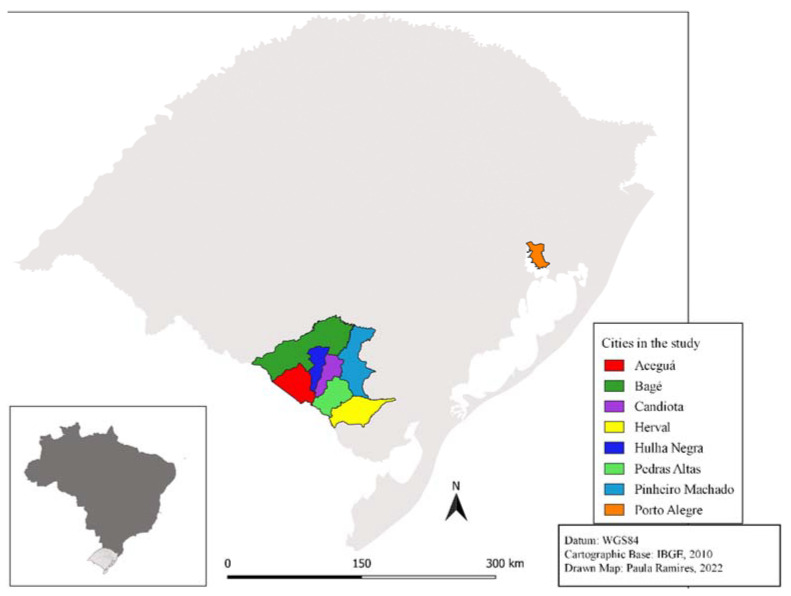
Map of the state of Rio Grande do Sul highlighting the cities involved in the study.

This study was carried out in seven cities in the state of Rio Grande do Sul, Brazil: Candiota (31°33′28″ S 53°40′22″ W; *n* = 128), where a coal-fired power plant and two coal surface-mining operations are situated, and six neighboring municipalities—Aceguá (31°52′ S 54°09′ W; *n* = 53), Bagé (31°19′51″ S 54°06′25″ W; *n* = 1549), Hulha Negra (31°24′14″ S 53°52′08″ W; *n* = 65), Pedras Altas (31°43′58″ S 53°35′02″ W; *n* = 21), Pinheiro Machado (31°34′40″ S 53°22′51″ W; *n* = 93), and Herval (32°01′26″ S 53°23′45″ W; *n* = 4) (Figure 1). These six municipalities are located in the Candiota region and are considered cities influenced by coal extraction and burning activities [27].

### 2.2. Analysis of Air Pollutants

Data on air pollutants—coarse particulate matter (PM_10_), sulfur dioxide (SO_2_), nitric oxide (NO) and nitrogen dioxide (NO_2_)—were extracted from automatic monitoring stations installed in three cities in the region: Candiota (city where coal extraction and burning activities are carried out), Aceguá (city located west of Candiota) and Pedras Altas (city located east of Candiota).

### 2.3. Data Collection

This epidemiological study was cross-sectional and retrospective. The data were obtained from the Declarations of Live Births (DLBs) of all children born during the year of 2013 to mothers residing in the study area. DLB information was also collected in hospitals from two neighboring municipalities, where some of those mothers eventually gave birth to their children. The DLB is a tool whose objective is to provide important information on the characteristics of live births, together with data on the mother and pregnancy, throughout the Brazilian territory. This information was collected to adequately assess the evolution of maternal and newborn care and health based on statistical, sociodemographic and epidemiological data. Since it was implemented by the Ministry of Health, the government keeps the data and health professionals (nurses, doctors) are responsible for completing the DLB after the birth of the child.

All ethical principles laid down by the National Health Council were respected, and the Ethics Committee in Health Research from the Universidade Federal do Rio Grande (CEPAS/FURG) approved this study under protocol nº 036/2013.

### 2.4. Variables

Maternal, pregnancy and neonatal outcomes were collected, such as first month of prenatal care (first trimester, second trimester, third trimester), prenatal care (inadequate, <6 consultations; adequate, ≥6 consultations) and number of previous pregnancies and abortions. Pregnancy and birth outcomes included gestational age (preterm, <37 weeks; term, ≥37–41 weeks and 6 days; post-term, >42 weeks), 5th minute Apgar score (score ≤ 6, score ≥ 7); birth weight (≤2499 g, 2500–2999 g, ≥3000 g); and presence of congenital disorders (no, yes).

### 2.5. Data Analysis

The data were double-digitized and checked into EPIINFO software, and the analysis was subsequently conducted using STATA 10.0. First, the prevalence of each variable and outcome was calculated. To estimate the crude and adjusted prevalence ratios and their respective confidence intervals (95%) as well as the *p*-values, Poisson regression was performed with robust variance estimates, according to hierarchical levels (1st Level—maternal sociodemographic: maternal education, matrimonial status, age and skin color and environmental exposure to PM_10_, NO_2_ and SO_2_ in the first trimester of pregnancy (using data from the Candiota air quality monitoring station); 2nd Level—maternal housing; 3rd Level—reproductive history: previous fetal losses/abortions and pregnancies, birth type; 4th Level—prenatal care and birth conditions: prenatal consultations and initiation of prenatal care; 5th Level—birth type, gestational age). To avoid confounding factors, the variables that presented *p* ≤ 0.2 were maintained in the model until the end, even though they lost their significance with the introduction of other variables of a lower hierarchical level. In the unadjusted and adjusted analyses, only children born from single pregnancies were included (*n* = 1910), while in descriptive analysis all births were included (*n* = 1950). In all analyses, values were considered significant when *p* < 0.05.

## 3. Results

Detailed information on the levels of air pollutants monitored in the region is described in Table 1. The annual averages of PM_10_ exceeded the Brazilian limits at the Candiota station and at the Aceguá station. The other pollutants did not have averages above the limits for annual averages. Even so, sporadic daily episodes exceeded the daily limits provided for in the legislation.

This study evaluated 1950 births among the seven municipalities from the coal mining region. As shown in Table 2, most mothers were 20–34 years old, self-reported Caucasian, had more than eight years of education and were not married. The majority of mothers reported previous pregnancies (55.2%) and 26.8% reported fetal loss or abortion.

Most mothers began their prenatal care in the first semester (77.4%), and had adequate prenatal care (68.6%). The majority of newborns were born by cesarean (68.6%) and were boys. Despite the low prevalence of negative outcomes (congenital disorders), 11.6% of newborns were preterm, 1.1% had 5th minute Apgar score of <6, 9.5% were of low birth weight and 68.3% of weight ≥3000 g at birth. Less than 1% had congenital disorders (Table 3).

Stratified analysis showed significant differences between the municipalities: Candiota had a high prevalence of births at <37 weeks (14.9%; Table 4). When stratified birth weight was analyzed a higher prevalence of weight of <2500 g was found in Pedras Altas (14.3%).

Table 5 shows the prevalence ratios and 95% confidence intervals for the variables used in unadjusted and adjusted models. The dependent variable was preterm birth. In both analyses, previous fetal loss/abortions, prenatal care and birth type had statistical significance. After adjustment for the covariates previous fetal loss/abortions and birth type, the prevalence ratios were increased significantly. Mothers with inadequate prenatal care had a two-fold higher risk to their newborn of preterm birth. First trimester PM_10_ exposure was significantly associated with preterm birth only in the adjusted model. The other variables investigated did not show significant associations with preterm birth, including NO_2_ and SO_2_.

As shown in Table 6, Black or mixed mothers had higher risk of newborns with low birth weight, as well as inadequate prenatal care and late start to prenatal care. Mothers with inadequate prenatal care had a more than two-fold higher risk of preterm birth. As with the effect on preterm birth, exposure to PM_10_ in the first trimester was significantly associated with low birth weight only in the adjusted model. Low birth weight did not show associations with the other variables investigated, including NO_2_ and SO_2_.

## 4. Discussion

Several factors can determine maternal and child health, such as biological, social and environmental conditions. This study evaluated maternal and newborn outcomes among 1950 births from seven municipalities situated in the largest Brazilian coal mining region, in addition to evaluating the risk of preterm birth and low birth weight. This region has received special attention in environmental health [11,23,24,25,31,32,33] and epidemiological studies [2,28,29,34,35] because it is a socioeconomically and environmentally vulnerable region.

Environmental and social vulnerability renders the population more susceptible to environmental pollution [27,36]. Maternal sociodemographic characteristics have important influences on perinatal or neonatal outcomes, especially in coal mining. Higher levels of air pollutants are typically more common in areas with lower socioeconomic levels [37]. Most of the mothers studied self-reported as Caucasian, which is protective against low birth weight. According to Casey et al. [38] women of all other races/ethnicities tend to experience higher levels of air pollution and other environmental hazards prenatally, which are correlated with adverse birth outcomes.

Abortion is considered the most common and severe complication of pregnancy. In Brazil the incidence range is 13–28% [39]. Abortion is a multifactorial etiology and involves both biological and environmental aspects. Air pollutants may represent a risk factor for spontaneous abortion and stillbirth [40]. This study observed a high prevalence of fetal loss or abortion; reinforcing our results, a study from Croatia found high frequencies of spontaneous abortion near open coal power plants [41]. Additionally, our study pointed out that previous fetal loss or abortion was a risk factor for preterm birth.

In this study, a significant proportion of mothers had later initiation of prenatal care and an insufficient number of visits compared to other coal mining areas [17]. Prenatal care should be through the incorporation of good practices in order to prevent maternal and neonatal mortality [42]. It is estimated that at least 10% of infant deaths could be prevented if all pregnant women had at least six prenatal consultations and were subjected to basic clinical and laboratory tests [43]. In addition, in this population, late initiation of prenatal care was a risk factor for preterm birth and low birth weight, emphasizing the importance of health care in a vulnerable population.

The prevalence of cesarean births in the study was much higher than that recommended by the WHO (10–15%). There are studies that show a high prevalence of C-sections in Brazil, and in the cities of Rio Grande do Sul State. Marmitt et al. [44], in their study in the city of Rio Grande, pointed that in the public sector, most women who had C-section were primiparous and older than 20 years of age, similar to the profile we identified in our study. In the private sector, those authors mentioned that no maternal characteristics interfered in the very high prevalence of C-sections. Eufrásio et al. [45] also explain that South Region, Brazil (which includes the states of Paraná, Santa Catarina and Rio Grande do Sul) is richer and more developed than the other regions in the country and this factor may explain why more C-sections are conducted in this region. They also mention that the higher prevalence of C-sections is accompanied by higher prevalence of preterm births, as we observed. The literature reports higher neonatal morbidity and mortality from cesarean delivery [3,46]. In this study, cesarean delivery increased the risk of preterm birth. The frequency of preterm birth found was higher than the national rate [42] and the rates in other coal mining areas worldwide [14,38]. It is important to highlight that the risk of adverse health effects from air pollution is particularly high for preterm births, given the vulnerability of their immature organ systems to outside influences [4,16,17].

The prevalence of congenital disorders was similar to that of the Rio Grande do Sul State [47] and higher than observed in a nearby coal mining region in the same county (0.4%; [48]). In Northern China, a relatively high risk of neural tube defects was reported among residents of a coal mining region [49]. Additionally, Ahem et al. [16] showed that up to 6 km away from coal mines there was still a high prevalence of neural tube defects compared to more distant areas, highlighting the influence of coal pollutants on newborn outcomes.

Approximately 12% of Brazilian births are premature [50]. Stratified analysis showed a higher prevalence of preterm births in Candiota city than in the other six neighboring municipalities. Casey et al. [39] observed a reduction in the frequency of preterm birth following the closure of coal and oil power plants. These results suggest that coal pollutants may increase the risk of preterm delivery, and that clean energy sources could bring benefits to the exposed population. Furthermore, considering that Candiota presented the highest median PM_10_ concentrations (Table 1), and that this pollutant has already been related to preterm births in recent studies [51,52,53], this is also a determinant that may be linked to this specific finding. Bigliardi et al. [30] observed that Candiota, as the host city of the coal exploration activities, showed a higher prevalence of obstructive ventilatory disorder cases than other cities in the region, with approximately 20% of its residents having altered lung function. Maternal respiratory diseases, whether acute or chronic, during the gestational period have also been associated with an increased risk of fetal prematurity [54].

In the region, special attention should be given to particulate matter, as it has been associated with low birth weight and premature births. In addition, while concentrations of NO_2_ and SO_2_ are often below the limits of environmental legislation [55], the average concentration of PM_10_ exceeds legal limits and those recommended by the WHO [56].

The prevalence of low birth weight (9.5%) did not reach the level considered a public health problem (15%; [57]). On the other hand, a recent systematic review involving studies conducted only with the Brazilian population showed high rates of excess birth weight (4.1 and 30.1%) depending on the classification criteria used and the region studied. However, in the region of the present study an unexpectedly high prevalence of high birth weight was observed (68.3%), which may be related to the nutritional status of the mothers and can be addressed in further studies. In fact, the unpublished information brought in this study, including the health data of each municipality, can help health services in planning and actions aimed at maternal and child health.

The study has some limitations, as the data were collected from DLB forms, which can show errors in completion. Additionally, these forms do not contain lifestyle information such as smoking, alcohol consumption, maternal weight and family income, which are important predictors of maternal and neonatal outcomes. Furthermore, the cities studied had different numbers of records because many births were not in the city of residence of the mothers, due to the precarious health conditions.

## 5. Conclusions

Of the total of 1950 annual births in the region, 11.6% were premature and 9.5% of newborns had low birth weight (<2500 g). Skin color, previous miscarriage, type of delivery and prenatal care were associated with these two outcomes (prematurity and low birth weight). In addition, higher levels of PM_10_ during the first trimester of pregnancy were associated with the outcomes studied. The sum of socioeconomic and environmental vulnerability in this population exposes it to the risks of unfavorable health outcomes.

## Figures and Tables

**Table 1:** Information on the concentrations (µg/m^3^) of air pollutants in the study region.

	Candiota Station				Pedras Altas Station				Aceguá Station			
	NO	NO_2_	SO_2_	PM_10_	NO	NO_2_	SO_2_	PM_10_	NO	NO_2_	SO_2_	PM_10_
Minimum	0	0.19	0.82	5.00	0.00	0.75	0.82	3.00	0.00	1.32	0.82	2.00
25th Percentile	2.33	4.33	5.32	18.00	1.72	3.20	7.77	11.00	1.72	3.01	9.00	15.75
Median	3.19	6.21	7.77	24.00	1.96	4.14	9.82	16.00	1.96	4.14	10.63	21.00
75th Percentile	4.79	9.03	12.27	32.00	2.09	5.08	11.04	22.00	2.21	5.27	12.68	27.00
Maximum	72.53	52.88	264.62	116.0	16.45	41.96	36.40	52.00	8.96	29.54	24.54	57.00
Mean	4.19	7.66	13.46	26.71	2.01	4.33	9.80	17.49	1.99	4.56	10.63	22.09
Std. Deviation	3.44	5.34	20.56	12.90	0.64	2.18	4.05	8.66	0.46	2.21	3.56	9.40

NO, nitric oxide; NO_2_, nitrogen dioxide; SO_2_, sulfur dioxide; PM_10_, coarse particulate matter.

**Table 2:** Maternal demographics from a coal mining area, Brazil.

	*n*	%
Age		
≤19 years	373	19.1
20–34 years	1320	67.7
≥35 years	257	13.2
Skin color declared +		
Caucasian	1424	73.1
Black or mixed	505	25.9
Education level +		
1 to 3 years	84	4.3
4 to 7 years	703	36.1
≥8 years	1147	58.8
Married +		
Not	1355	69.5
Yes	580	29.7
Previous pregnancies +		
Zero	868	44.5
1 gestation	544	27.9
≥2 gestations	533	27.3
Previous fetal loss/abortions *+		
Zero	791	73.1
≥1 loss	290	26.8

* *n* = 1081; 869 pregnant women had no previous gestation. + Some variables had lost information; Skin color declared was the variable with the most information lost with 100 cases missing.

**Table 3:** Maternal and neonatal outcomes and pre-natal assistance in cities in a coal mining area, Brazil.

	*n*	%
Initiation of prenatal care +		
1–3 month	1510	77.4
4–6 month	300	15.4
7–9 month	40	2.1
Prenatal care +		
Inadequate	254	13.0
Adequate	1651	84.7
Birth type +		
Vaginal	611	31.3
Cesarean	1337	68.6
Gender of the newborn		
Male	980	50.3
Female	970	49.7
Gestational age +		
Preterm	223	11.6
Term	1684	86.4
Post-term	31	1.4
5th minute Apgar score +		
≤6	21	1.1
≥7	1920	98.5
Birth weight (g) +		
≤2499	184	9.5
2500–2999	433	22.2
≥3000	1332	68.3
Congenital disorders +		
No	1902	97.5
Yes	17	0.9

+ Some variables had lost information; Initiation of prenatal care was the variable with the most information lost, with 100 cases missing.

**Table 4:** Gestational age and birth weight by city.

	Total	Candiota	Aceguá	Bagé	Herval	Hulha Negra	Pedras Altas	Pinheiro Machado	*p*-Value
	*n* (%)	*n* (%)	*n* (%)	*n* (%)	*n* (%)	*n* (%)	*n* (%)	*n* (%)	
Gestational age (weeks)									<0.01
≤31	30 (1.5)	-	03 (5.7)	22 (1.4)	01 (2.4)	01 (1.5)	01 (4.8)	02 (2.2)	
32–33	35 (2.0)	02 (1.6)	-	28 (1.8)	01 (2.4)	01 (1.5)	01 (4.8)	02 (2.2)	
34–36	158 (8.1)	17 (13.3)	03 (5.7)	128 (8.3)	03 (7.3)	04 (6.2)	-	03 (3.2)	
37–41	1684 (86.4)	102 (79.7)	44 (83.0)	1356 (87.5)	35 (85.4)	55 (84.6)	16 (76.2)	76 (87.0)	
≥42	31 (1.6)	06 (4.7)	03 (5.7)	10 (0.6)	01 (2.4)	03 (4.6)	03 (14.3)	05 (5.4)	
Birth weight (g)									<0.01
≤999	09 (0.5)	-	02 (3.8)	05 (0.3)	01 (2.4)	01 (1.5)	-	-	
1000–1499	16 (0.8)	-	-	14 (0.9)	-	-	02 (9.5)	-	
1500–2499	159 (8.2)	13 (10.2)	03 (5.7)	129 (8.3)	01 (2.4)	0,2 (3.1)	01 (4.8)	10 (10.8)	
2500–2999	433 (22.2)	31 (24.2)	09 (17.0)	350 (22.6)	08 (19.5)	08 (12.3)	04 (19.0)	23 (24.7)	
3000–3999	1215 (62.3)	80 (62.5)	31 (58.5)	958 (61.8)	30 (73.2)	49 (75.4)	12 (57.1)	55 (59.1)	
≥4000	117 (6.0)	04 (3.1)	08 (15.1)	93 (6.0)	01 (2.4)	05 (7.7)	02 (9.5)	04 (4.3)	

Gestational age was the variable with more lost information (12 lost information).

**Table 5:** Unadjusted and adjusted prevalence ratios (with 95% confidence limits) for pre-term birth among a coal mining area in Brazil.

	Unadjusted	*p*-Value	Adjusted	*p*-Value
Previous fetal loss/abortions		0.02		0.01
Zero	1.00		1.00	
≥1 loss	1.57 (1.09–2.26)		1.58 (1.10–2.27)	
Prenatal care		<0.01		<0.01
Adequate	1.00		1.00	
Inadequate	2.57 (1.93–3.42)		2.39 (1.62–3.52)	
Birth type		0.01		0.01
Vaginal	1.00		1.00	
Cesarean	1.52 (1.11–2.08)		1.87 (1.16–3.02)	
PM_10_ in the first trimester of pregnancy	0.99 (0.95–1.04)	0.77	1.31 (1.11–1.55)	<0.01

**Table 6:** Unadjusted and adjusted prevalence ratios (with 95% confidence limits) for low birth weight among a coal mining area in Brazil.

	Unadjusted	*p*-Value	Adjusted	*p*-Value
Skin color declared		<0.01		0.01
Caucasian	1.00		1.00	
Black or mixed	1.52 (1.11–2.08)		1.53 (1.12–2.10)	
Prenatal care		<0.01		<0.01
Adequate	1.00		1.00	
Inadequate	3.06 (2.24–4.19)		2.94 (2.14–4.05)	
Initiation of prenatal care		0.03		0.47
1–3 month	1.00		1.00	
4–6 month	1.64 (1.14–2.35)		1.01 (0.67–1.50)	
7–9 month	1.03 (0.34–3.10)		0.51 (0.16–1.68)	
PM_10_ exposure in the first trimester of pregnancy	1.01 (0.96–1.06)	0.75	1.27 (1.06–1.52)	0.01

## Data Availability

The datasets used and/or analyzed during the current study are available from the corresponding author on reasonable request.
